# Epidemiological *evaluation* of *Neospora caninum* in dairy animals

**DOI:** 10.1590/1984-3143-AR2022-0104

**Published:** 2023-05-15

**Authors:** Irina Ikonnikova, Yessengali Ussenbekov, Vladimir Domatskiy, Yuliya Lazareva

**Affiliations:** 1 Department of Medical Computer Science and Statistics, I. M. Sechenov First Moscow State Medical University (Sechenov University), Moscow, Russian Federation; 2 Department of Obstetrics, Surgery and Biotechnology of Animal Reproduction, Kazakh National Agrarian Research University, Almaty, Republic of Kazakhstan; 3 Department of Infectious and Invasive Diseases, Federal State Budgetary Educational Institution of the Higher Education “Northern Trans-Urals State Agrarian University”, Tyumen, Russian Federation; 4 Department of Biology and General Genetics, I. M. Sechenov First Moscow State Medical University (Sechenov University), Moscow, Russian Federation

**Keywords:** neosporosis, Neospora caninum, cattle, seropositivity, seroprevalence

## Abstract

The purpose of the study is to explore the epidemiological information concerning the spread of neosporosis in the Moscow region (Russian Federation) and Almaty region (Republic of Kazakhstan). The study is conducted in 2019 in the Moscow region (Russian Federation) and Almaty region (Republic of Kazakhstan). The study sample includes 800 cows (400 animals at each of the two locations), 100 at each of the 4 cattle farms in the Moscow region and 100 at each of the 4 cattle farms in the Almaty region. There were more seropositive cows on the rest of farms as compared to farm No. 1: 1.9 times more on farm No. 2 (p ≤ 0.01), 2.4 times more on farm No. 3 (p ≤ 0.001), and almost 4 times more on farm No. 4 (p ≤ 0.0001). In terms of the abortion rates, the maximum difference between the farms was 5-fold in the Moscow region (p ≤ 0.0001) and 3-fold in the Almaty region (p ≤ 0.001). Positive correlations exist between all the studied parameters (the proportion of seropositive animals, the proportion of seroprevalent animals, the abortion rate, and the stillbirth rate). The results of the study are mainly extremely valuable for the global economy, because the Republic of Kazakhstan and the Russian Federation occupy an important place in the export of meat and dairy products.

## Introduction

In today’s livestock breeding, neosporosis is a big problem, since one of the main consequences of this disease is an increased risk of abortion, birth of non-viable calves, or congenital brain malformations (Guido et al., 2016). Nevertheless, the available information suggests that detrimental effects of neosporosis depend on a number of factors, including gestational age. If infection occurs during early gestation, the only outcome is abortion. If cows become infected at mid to late gestation, they give birth to calves whose cells contain *Neospora caninum* (i.e., calves are congenitally infected with *Neospora caninum)* (Reichel et al., 2014).

The importance of *Neospora caninum* for animal husbandry is not limited to the awareness of an increased risk of abortion or birth of infected calves. Numerous cases of decreased meat and dairy cattle production have been recorded for the infected cattle (Ansari-Lari et al., 2017). The above describes horizontal transmission; in addition, intrauterine transmission can occur. In this case, about 80% of newborn calves become parasite carriers. Further, this generation can transmit the parasite to the next one, now vertically. *Neospora caninum* can be maintained in the cattle herds through generations (Almería et al., 2017). Based on the new information, cattle vaccination does not mean complete exclusion of uterine infections (Novoa et al., 2020). Until lately, the only way to prevent the spread of neosporosis was not to breed heifers born to infected cows. This affected livestock breeding and resulted in significant losses caused by exclusion of genetically valuable animals (Dubey and Schares, 2011). In another study, an experiment involved transferring embryos from infected animals to healthy dams. This excluded vertical transmission of *Neospora caninum* (Weston et al., 2012). The reported cases of the cattle infection with *Neospora caninum* led to the conclusion that dairy cattle was affected to a greater extent than the beef one (Ansari-Lari et al., 2017).

The stages of neosporosis are characterized by different durations (Lefkaditis et al., 2020). After being introduced into the stomach, *Neospora caninum* enters the small intestine and subsequently converts into tachyzoites. The latter divide rapidly and disseminate throughout the body via the bloodstream. The central nervous system, the uterus, as well as myocytes, hepatocytes and nephrocytes are affected in the first place. This is the reason why reproductive and nervous organs of the infected animal are targeted first. The affected cells of the intermediate host are used by the parasite as an energy source. In cells, the parasite replicates intensively for up to 1 week. Upon this period, the host’s immune system activates and launches the next stage of neosporosis - cysts formation. Interestingly, cysts accumulate not only in the muscle tissues, but also in the nerve cells and fetal membranes. This stage (bradyzoite) precedes the direct parasite’s invasion of the definitive host’s (i.e. the dog’s) organism.

It is undeniable that neosporosis has a negative economic impact on the livestock production (Demir et al., 2020). According to various sources, its prevalence varies greatly depending on the region (Ostrikov et al., 2018, 2019). Thus, a quarter of all cattle (24%) are affected in Latin America, 18% in Asia, and up to 14% in Europe and Africa who boast of the lowest infection rate (Reichel et al., 2013). For many countries around the world that have industrial-scale dairy cattle farms, neosporosis is the main cause of abortions and reduced dairy production.

Despite strong awareness of neosporosis worldwide, the Russian Federation and Republic of Kazakhstan can serve as convenient model regions for neosporosis research, since no information on the state of the problem in these countries has been presented in the leading western journals on veterinary and clinical veterinary. The only exception was two works that mention *Neospora caninum* isolated from the sheep raised in the territory of the Republic of Kazakhstan (Hui et al., 2013; Olias et al., 2011). This determines the relevance of this work. On average, 8% of abortions in dairy cattle and 9% of their stillbirths are attributed to *Neospora caninum* (Reichel et al., 2015). This research is focused on dairy cattle, as they are more susceptible to *Neospora caninum* infection. Data presented in this study can help improve the epidemiological situation in the studied regions; note that Russia and Kazakhstan are crucial suppliers of dairy and meat products A large number of animals infected with neospores (cattle in particular) will produce less offspring or the offspring will not be viable, causing significant economic damage. Thus, it is vital to identify epidemiologically disadvantaged farms and reasons behind the high incidence of disease among cattle.

This study aims to present a comparative analysis of neosporosis incidence in two model regions: Moscow region (Russian Federation) and Almaty region (Kazakhstan). The purpose of the study is to assess neosporosis prevalence on animal farms located in the Moscow region (Russian Federation) and Almaty region (Republic of Kazakhstan). The authors assume that animal farms that are able to limit the access of definitive hosts of *Neospora caninum* (dogs) to fetal membranes and infected animal tissues will have a lower percentage of infected cows. In addition, the authors believe that there are pronounced correlations between seropositivity, seroprevalence, the abortion rate, and the stillbirth rate, which manifest themselves in different ways in the examined regions, depending on the current epidemiological situation. The objectives of the study are the following: a) to assess the prevalence of neosporosis in the Moscow region (Russian Federation); b) to assess the prevalence of neosporosis in the Almaty region (Republic of Kazakhstan); c) to compare the prevalence rates among dairy cattle kept at two different locations and identify the causes of the high prevalence rates; d) to perform serological tests in the cow herds with a high prevalence of neosporosis.

The choice of cows for the study was due to the value and importance of epidemiological indicators because these animals provide meat and dairy products in all countries of the world.

## Methods

### Sample

The study is conducted in 2019 in the Moscow region (Russian Federation) and Almaty region (Republic of Kazakhstan). Serum samples are collected from 400 cows and aborting heifers in the Moscow region (100 animals kept at each of the 4 cattle farms) and from the equal number of cows and aborting heifers in the Almaty region (100 animals kept at each of the 4 cattle farms). The animals are selected randomly on each of the farms. The average distance between farms was 15 km in the Moscow region and 12 km in the Almaty region. There was no exchange of semen samples and young claves between farms during the observation period. Farms like these were chosen to exclude the risk of infection being transmitted from one farm to another. There were no animals present on these farms that could be part of the *Neospore* life cycle. Manure samples were not taken. Stillborn fetuses were not tested for *Neospores* infection.

Each farm holds 500 cows, of which 20% were selected for analysis. The selection criteria were the average abortion rate of 3% or higher and the average frequency of stillbirths more than 4% among neospore-infected cows. All animals that met these criteria were isolated from the heard.

Milk production enterprises with 600 to 800 cows and even larger producers normally use a flow shop system to organize milk production and herd reproduction, which requires the herd to be divided into shops based on the physiological state of the animals. The sorting criteria include cows giving birth (maternity ward), lactation, milk production, and cows in their dry period (these are usually kept loose). It is permissible to combine the lactation and milk production shops.

In smaller capacity farms like those with 500 heads, heifers and more mature cows are kept separately within the maternity ward. If the straw yard system is chosen, there also can be a separately kept group of dry cows.

### Study design

When neosporosis is suspected, the recommendation is to perform histological and immunological examination of aborted fetuses and stillborn calves. For adult animals, serological methods are used. If within 6 weeks of the experiment, abortions are defined as taking place on the 100-260th day of pregnancy in more than 15% of cows, then the examination should focus solely on animals in the second half of pregnancy (the barren ones are not taken into account). If the number of aborted animals becomes lower and more time passes between the abortions, then it the entire herd should get examined.

Only those farms are involved which reported prior stillbirths or abortions. The study does not include animals with other serious chronic diseases that could affect the mortality of adult cows or calves - cattle plague, chronic diarrhea, mycoplasmosis.

### The ethical statement

This study meets the international ethical and moral standards for the treatment of animals. The study is approved at a meeting of the Ethics Committee at the Kazakh National Agrarian Research University (Protocol no. 34-A934 dated from 02/02/2019). To ensure anonymity and confidentiality, the names of the livestock farms are not disclosed, and the farms at both locations are mentioned in this paper under numbers from 1 to 4. The study includes farms with the abortion rates of 3.0-5.0% (based on the statistical data).

### Methods

Each of the examined animals had its blood samples taken from the tail vein. The samples were taken no later than three weeks before calving and no earlier than 3 weeks after calving. Blood was collected in the morning before feeding. It is best to sample venous blood with the help of vacuum-containing systems. This approach has its advantages, the main of which is that the blood enters directly into a closed tube. The vacuum-containing system is a closed two-component system with a syringe and a special needle. The samples were stored in a refrigerator at a temperature of 2-5 °C for 1 day before being sent for testing. After blood samples had been collected, the serum was extracted by leaving the blood samples undisturbed at a temperature of 25-30 °C for 1 hour. The resulting blood clot was separated from the serum, which was poured into a special separate container. After that, serum samples were sent to the laboratory, where they were stored at a temperature of -20 °C.

Whole blood, plasma and serum were stored in a refrigerator (2-4°C) for a short period of time. It case of serum, the long-term storage would require a lower temperature (-20°C). Blood serum was collected using the sludge method. To achieve blood coagulation and serum separation, the blood-containing tubes were kept at 20-30°C or 37-38°C for 30 to 60 min; the blood clots were separated from the tube walls with a steel wire. The tubes were then kept at 4-10°C for 20 to 24 hours. The temperature regime matched to the temperature of the incubator. The laboratories were located in Moscow (to process samples from Russia) and in Almaty (to process samples from Kazakhstan). All samples in both laboratories were processed under a single protocol.

The presence of antibodies to *Neospora caninum* was investigated by enzyme-linked immunosorbent assay. During the assay procedure, IDEXX Neospora Ab Test kits (manufactured by IDEXX Laboratories Inc., USA) were used which are designed to detect antibodies to *Neospora caninum* in serum. The same kits and serum assay methods were applied at both locations in order to obtain uniform results. In order to analyze the results obtained, the interpreting software called xChek Assay Management System (manufactured by IDEXX Laboratories Inc., USA) was used.

The PCR protocol was as follows: biomaterial (cerebrospinal fluid or CSF) is taken before the contrast medium for myelography is injected or not earlier than 5 days after the procedure. The procedure requires general anesthesia. The animals were not fed on the day of CSF collection. CSF was placed into an EDTA tube. After the tube has been filled with CSF, it was carefully inverted 6-7 times to mix the material with the anticoagulant. The tube was then left frozen at a vertical position at -17-23°С and kept like that for a month. Each tube was labeled with the farm name, animal name, and sampling location. The transportation temperature was -17-23°С. The minimum volume of the sample was 1 ml. CSF samples were centrifuged on Eppendorf (14 thousand rpm). If after centrifugation the specimen became transparent then its turbidity before centrifugation was defined as stemming from the presence of formed elements. If it remained turbid, then the main reason was the presence of microorganisms. Samples were collected in BSL2 containers. The PCR procedure involved the use of *Neospore ampli-test primers* (Russia).

### Statistical analysis

The data obtained were entered into Excel 2016 (Microsoft Inc., USA) database. Subsequently, the data were statistically processed using Statistica 7.0 (StatSoft, USA). The Student’s t-test was applied to identify significant differences between the two independent samples. The proportions of cattle susceptible to neosporosis (seropositive animals) and cattle suffering from neosporosis (according to the test results, seroprevalent animals) were shown as a percentage. In addition, the Pearson correlation was calculated between the studied parameters. The minimum level of confidence was p ≤ 0.05.

## Result

The proportions of seropositive and seroprevalent animals differed significantly between the farms in the Moscow region and Almaty region (Tables 1 and 2). In the Moscow region, the smallest proportion of seropositive animals was seen on farm No. 1.

**Table 1:** The proportions of seropositive and seroprevalent animals (as a % of the total herd size) on 4 cattle farms located in the Moscow region.

**Farm number**	**The proportion of** **seropositive animals**	**The proportion of** **seroprevalent animals**
Farm No. 1	17	15
Farm No. 2	33	32
Farm No. 3	41	39
Farm No. 4	65	62

**Table 2:** The proportions of seropositive and seroprevalent animals (as a % of the total herd size) on 4 cattle farms located in the Almaty region.

**Farm number**	**The proportion of** **seropositive animals**	**The proportion of** **seroprevalent animals**
Farm No. 1	54	51
Farm No. 2	67	63
Farm No. 3	78	74
Farm No. 4	93	91

There were more seropositive cows on the rest of farms as compared to farm No. 1: 1.9 times more on farm No. 2 (p ≤ 0.01), 2.4 times more on farm No. 3 (p ≤ 0.001), and almost 4 times more on farm No. 4 (p ≤ 0.0001). This meant that animals susceptible to infection were the most numerous on farm No. 4, which predetermined the subsequently obtained variables. The smallest number of seroprevalent animals was seen on farm No. 1. There were more seroprevalent cows on the rest of farms as compared to farm No. 1: 2 times more on farm No. 2 (p ≤ 0.01), 2.6 times more on farm No. 3 (p ≤ 0.01), and 4 times more on farm No. 4 (p ≤ 0.0001). Correlations have been discovered between these parameters (0.89, р ≤ 0.03). It follows from the above that seropositivity rate defines the risk of neosporosis (seroprevalence). Still, it is worth mentioning that the share of seroprevalent animals was slightly below the share of seropositive ones.

The comparative analysis of PCR and ELISA findings in the Moscow region revealed that PCR was generally 3-6% more effective in identifying seropositivity and seroprevalence (Table 1). Even though PCR tests had a higher detection rate, the within-farm differences were not significant (p ≥ 0.05).

In the Almaty region, seropositivity rates were even higher (Table 2). Thus, the minimum rate seen on farm No. 1 was practically the same as that on farm No. 1 in the Moscow region (p ≤ 0.05). Farm No. 2 in the Almaty region had 1.2 times more seropositive animals (p ≤ 0.05) than farm No. 1 (Table 2). There were 1.4 times more seropositive animals on farm No. 3 (p ≤ 0.05), and 1.7 times more of them on farm No. 4, which was the maximum number (p ≤ 0.01).

The share of seroprevalent animals differed significantly between the farms located in the Almaty region. The smallest share of seroprevalent animals was seen on farm No. 1, with 1.2 times as many on farm No. 2 (p ≤ 0.05), 1.4 times as many on farm No. 3 (p ≤ 0.05), and 1.8 times as many on farm No. 4 (p ≤ 0.01). Thus, one of the farms had the maximum number of infected animals, and it was the farm which recorded the maximum number of seropositive animals (Pearson’s correlation 0.81). In the Almaty region, the differences between farms related to the proportion of seropositive animals and proportion of seroprevalent animals, were slighter than in the Moscow region (p ≤ 0.01 for the difference between the two locations). This could be explained by the fact that the situation with the epidemic of neosporosis was more threatening in the Almaty region, where any and all of the examined farms registered high proportions of seropositive and seroprevalent animals.

Similarly, PCR tests appeared to be more effective than ELISA, with a 3 to 6% higher detection rate of seroprevalence and seropositivity. The differences were not significant (p ≥ 0.05). Hence, PCR is a better option, but ELISA would be good as well.

Due to neosporosis outbreak, there was a high abortion rate in the cattle herds held in the Almaty region as compared to the Moscow region (Table 3). When the biggest difference between the farms in the Moscow region was 5 times (the difference between farm No. 1 and farm No. 4, p ≤ 0.0001), it was 3.0 times for the Almaty region (the difference between farm No. 1 and farm No. 4, p ≤ 0.001).

**Table 3:** Comparison of abortion rates (%) on farms located in the Moscow region and Almaty region.

**Farm number**	**Abortion rates on farms located in the** **Moscow region, %**	**Abortion rates on farms located in the** **Almaty region, %**
Farm No. 1	3	13
Farm No. 2	7	16
Farm No. 3	9	20
Farm No. 4	15	39

On farm No. 2 in the Moscow region, the proportion of abortions was 2.3 times higher than on farm No. 1 (p ≤ 0.01). In the Almaty region, the difference was 1.2 times (p ≤ 0.05). On farm No. 3 in the Moscow region, the proportion of abortions was 3 times higher (p ≤ 0.001), in the Almaty region - 1.5 times higher (p ≤ 0.05). Obviously, the number of abortions did not increase in the Almaty region as sharply as in the Moscow region. The tendency resembles the one described above in relation to the data presented in Tables 1 and 2. This was also associated with large numbers of infected animals (correlation 0.91 for the Moscow region and 0.84 for the Almaty region).

Stillbirths occurred with similar frequency as abortions (Table 4). In the Moscow region, the difference between the proportion of stillborn calves on farms No. 1 and 4 was 4-fold (p ≤ 0.0001), in the Almaty region - 3.1-fold (p ≤ 0.001).

**Table 4:** Comparison of stillbirth rates (%) on farms located in the Moscow region and Almaty region.

**Farm number**	**Stillbirth rates on farms** **located in the** **Moscow region, %**	**Stillbirth rates on farms** **located in the** **Almaty region, %**
Farm No. 1	4	17
Farm No. 2	8	24
Farm No. 3	11	31
Farm No. 4	17	57

The difference between farms No. 2 and 1 in the Moscow region was 2-fold (p ≤ 0.01), in the Almaty region - 1.4-fold (p ≤ 0.05). The difference between farms No. 1 and 3 in the Moscow region was 2.9-fold (p ≤ 0.001), in the Almaty region - 1.9-fold (p ≤ 0.01). Thus, the proportion of stillborn calves was slightly higher as compared to the proportion of abortions at both locations (p ≤ 0.05 for the difference between the two locations). Again, correlations existed between the proportions of infected animals on the one hand and proportions of stillbirths on the other hand (0.91 and 0.86 for the Moscow region and Almaty region, respectively).

Among neospora-positive cows in the Moscow region, 18% were aborted. By contrast, the aborted neospora-negative cows accounted for just 2%. In the Almaty region, it was 35 and 4%, respectively. Thus, a positive reaction to neosporosis carries a high risk of abortion.

Consequently, the Almaty region showed a more modest increase in all the assessed variables as compared to the Moscow region. Nevertheless, it was obvious that situation with neosporosis was more threatening in the Almaty region than in the Moscow region because a comparative analysis of these regions showed that in all surveyed farms, the Almaty region had higher rates of seropositive and seroprevalent animals.

## Discussion

The authors assume that animal farms that are able to limit the access of definitive hosts of *Neospora caninum* (dogs) to fetal membranes and infected animal tissues will have a lower percentage of infected cows. In addition, the authors believe that there are pronounced correlations between seropositivity, seroprevalence, the abortion rate, and the stillbirth rate, which manifest themselves in different ways in the examined regions, depending on the current epidemiological situation. The results showed that a relatively low infection level was observed in 3 out of 4 livestock farms in the Moscow region, while all 4 farms in the Almaty region had the levels of *Neospora caninum* infection ranging from high to very high. The data obtained correlate with the results of the previous research indicating that the level of neosporosis infection may vary from 25 up to 100% (Donahoe et al., 2015; Imre et al., 2012; Lassen et al., 2012). The values yielded in the current study are also within this range. According to other data, seroprevalence level usually ranges from 45 to 60% in individual herds (Malaguti et al., 2012; Mazuz et al., 2014). In the current study, seroprevalence level of the examined herds kept on 4 farms in the Almaty region significantly exceeded the above reference ranges, which indicated an outbreak of neosporosis in the region. While the average abortion rate is 7% and the average stillbirth rate is 9% (Piagentini et al., 2012), the current study demonstrated that the Almaty region reported abortions in almost half of the cases and saw stillborn calves in more than half of the cases.

It is known that late-term pregnancies are associated with higher risk for pregnancy loss (Safonov, 2022; Ventsova and Safonov, 2021). Thus, 3-4.5 months pregnant experience reproductive loss in 19% of cases, while after 4.5 months of pregnancy loss occurs in 80% of cases (Špilovská et al., 2015). Interestingly, aborting cows do not display signs of disease. However, fetuses may be mummified, born clinically normal, or autolyzed (Špilovská et al., 2015).

The frequency of antibodies is also quite variable, with antibodies detected in 5 to 55% of animals belonging to each of the examined herds (Liu et al., 2020). This fact indicates that definitive hosts (dogs) are extensively exposed to the infectious agent (*Neospora caninum*). They become infected by consuming raw meat supplied from slaughterhouses and are subsequently used by the parasite as a part of its lifecycle.

As for the economic effect, despite the fact that the United States has rather low levels of cattle infection (2-3%), solely California spends up to 35 million dollars per year to fight *Neospora caninum*. In general, this infection persists in 36 out of 50 states of the US (Reichel et al., 2013). In Australia, annual financial losses due to abortions, stillbirths, and reduced dairy and beef cattle production amount to no less than 100 million dollars (Reichel et al., 2013). For the two locations described in this paper (Russian Federation and Republic of Kazakhstan), the prevalence rates observed are radically different. The above indicates the need to explore other areas of concern in order to assess the total financial losses incurred by the countries due to neosporosis.

It is recommended to take at least primary prevention measures, which include keeping cattle away from dogs. Otherwise, it would be extremely difficult to apply basic hygiene standards within the farm area and make forecasts related the number of healthy animals on a farm, since a significant number of pregnancies would end in stillbirths or abortions (Moore et al., 2015). This is due to the fact that neosporosis is the main cause of abortions and stillbirths in cows, primarily in dairy herds, as it has been shown in the current study and in a number of other studies (Ribeiro et al., 2019; Adanir et al., 2015; Piagentini et al., 2012; Mazuz et al., 2014). Hence, neosporosis is an integral part of the modern animal husbandry the main problem of which is gynecologic and obstetric pathologies.

Thereby, further research is needed, including one in the Russian Federation and Republic of Kazakhstan. It should seek the ways to minimize horizontal transmission of *Neospora caninum* and develop effective prevention measures, including proper farm organization (dairy farms operation and models), monitoring of the quality of semen taken from breeding bulls for artificial insemination, and constant control of the number of seropositive and seroprevalent animals. All these measures should be taken in combination to make the *Neospora caninum* control strategy highly effective.

## Conclusion

The present paper shows a close relation between all the assessed parameters (the proportion of seropositive animals, the proportion of seroprevalent animals, the abortion rate, and the stillbirth rate). All these parameters reflect neosporosis epidemiology, which varies on the examined livestock farms. In this context, despite stronger positive relation between the proportions of seropositive and seroprevalent animals in the Moscow region (0.89 versus 0.81 in the Almaty region), general epidemiological situation is better in the Moscow region than in the Almaty region (at least on the four largest farms housing thousands of animals). There are also correlations between the abortion rates and the proportions of seroprevalent animals (0.91 versus 0.84), as well as between the stillbirth rates and the proportions of seroprevalent animals (0.91 versus 0.86), with higher values obtained for the Moscow region as compared to the Almaty region.

Thus, given the outbreak of neosporosis, the correlations may not fully reflect the actual situation. All the examined farms in the Moscow region, except for farm No. 4, applied the simplest methods of reducing the chances for dogs coming into contact with contaminated biomaterial (embryos, the remains of adult animals), i.e., they kept the dogs in the fenced area. No such precautions were taken in the Almaty region. This embraces the need for primary prevention (excluding contacts between dogs and biomaterial left from cattle) which will help to reduce horizontal transmission of the infection. By following these simple prevention measures the Almaty region will be able to reduce its high prevalence of neosporosis at least to the levels observed in the Moscow region.
